# Bacterial flora-typing with targeted, chip-based Pyrosequencing

**DOI:** 10.1186/1471-2180-7-108

**Published:** 2007-11-30

**Authors:** Andreas Sundquist, Saharnaz Bigdeli, Roxana Jalili, Maurice L Druzin, Sarah Waller, Kristin M Pullen, Yasser Y El-Sayed, M Mark Taslimi, Serafim Batzoglou, Mostafa Ronaghi

**Affiliations:** 1 Department of Computer Science, Stanford University, Stanford, CA 94305, USA; 2 Stanford Genome Technology Center, Stanford University, Palo Alto, CA 94304, USA; 3 Department of Obstetrics and Gynecology, Stanford University Medical Center, Palo Alto, CA 94305, USA

## Abstract

**Background:**

The metagenomic analysis of microbial communities holds the potential to improve our understanding of the role of microbes in clinical conditions. Recent, dramatic improvements in DNA sequencing throughput and cost will enable such analyses on individuals. However, such advances in throughput generally come at the cost of shorter read-lengths, limiting the discriminatory power of each read. In particular, classifying the microbial content of samples by sequencing the < 1,600 bp 16S rRNA gene will be affected by such limitations.

**Results:**

We describe a method for identifying the phylogenetic content of bacterial samples using high-throughput Pyrosequencing targeted at the 16S rRNA gene. Our analysis is adapted to the shorter read-lengths of such technology and uses a database of 16S rDNA to determine the most specific phylogenetic classification for reads, resulting in a weighted phylogenetic tree characterizing the content of the sample. We present results for six samples obtained from the human vagina during pregnancy that corroborates previous studies using conventional techniques.

Next, we analyze the power of our method to classify reads at each level of the phylogeny using simulation experiments. We assess the impacts of read-length and database completeness on our method, and predict how we do as technology improves and more bacteria are sequenced. Finally, we study the utility of targeting specific 16S variable regions and show that such an approach considerably improves results for certain types of microbial samples. Using simulation, our method can be used to determine the most informative variable region.

**Conclusion:**

This study provides positive validation of the effectiveness of targeting 16S metagenomes using short-read sequencing technology. Our methodology allows us to infer the most specific assignment of the sequence reads within the phylogeny, and to identify the most discriminative variable region to target. The analysis of high-throughput Pyrosequencing on human flora samples will accelerate the study of the relationship between the microbial world and ourselves.

## Background

Metagenomics enables the genomic study of microbial communities that are sampled directly from their environment, eliminating the need for isolating and cultivating specific microbes [1-3]. Metagenomic analyses of human flora samples [4] are a new type of assay with intriguing potential applications for the diagnosis and prediction of clinical outcomes [5]. Studies of human vaginal bacterium during pregnancy so far include the use of direct culture methods and conventional PCR studies of clinically suspected infectious microorganisms. Although infection and inflammation likely play a major role in the pathogenesis of preterm labor and delivery [6,7], these studies reveal only a fraction of the potential microorganic inhabitants. A comprehensive identification and catalog of these organisms will enable future investigators to target a defined population of species that may be correlated with preterm labor, premature rupture of amniotic membranes, chorioamnionitis, and other complications of pregnancy [8-12].

Metagenomics analyses will become increasingly practical as DNA sequencing costs fall dramatically with the advent of new technologies [13,14] including Pyrosequencing™ [15]. One challenge common to these revolutionary sequencing technologies is the short length of reads, which limits the amount of unique, discriminating sequence available within each read. Sequencing the 16S rRNA gene (16S rDNA) using conventional Sanger sequencing produces reads of at least 500 bp in length, which is sufficient to identify the precise source species for each gene [3]. In fact, though there is a danger of producing chimeras, the reads are often long enough that they can be assembled into near-complete 16S rDNA sequences [16]. Despite the promise of high-throughput technologies like Pyrosequencing, current versions produce short reads, making the accurate identification of the source of these reads a daunting task. One solution used chip-based Pyrosequencing targeted at a small variable region within the 16S rDNA to show that there exists a much greater variety of rare microorganisms than previously thought [17].

We describe a methodology for phylogenetic classification based on short, 16S rRNA gene sequence reads and apply the technique to reads obtained via high-throughput, chip-based Pyrosequencing of human vaginal flora samples during pregnancy. The resulting phylogenetic trees reveal the vast diversity of bacterial inhabitants seen in other studies, and will assist in future investigations of the link between microorganisms and pregnancy complications. Next, we examine the ability of our methodology to classify reads at different levels in the phylogeny and discuss limitations of our technique. Using simulations, we study the effect of read-length on our methodology to understand the consequence of using high-throughput Pyrosequencing instead of conventional technologies. Finally, we explore the effectiveness of isolating specific 16S variable regions using validated universal primers. Our methodology for analyzing short 16S rDNA sequence reads will enable the accurate and informative study of human flora samples using new, high-throughput sequencing technologies.

## Results and Discussion

### Methodology overview

Twelve samples from vaginal epithelial tissue and discharge from pregnant women in all three trimesters were collected. DNA extraction was performed, followed by target-specific PCR amplification of approximately 1500 bp of the 16S rDNA using universal primers. The products were subjected to nebulization and clonal amplification, followed by Pyrosequencing of six samples with the Genome Sequencer 20 system (454 Life Sciences). As a result, 100,000 to 200,000 sequence reads of 100 bp average length were obtained for each of the six samples (details are provided in Additional file 1).

**Figure 1:**
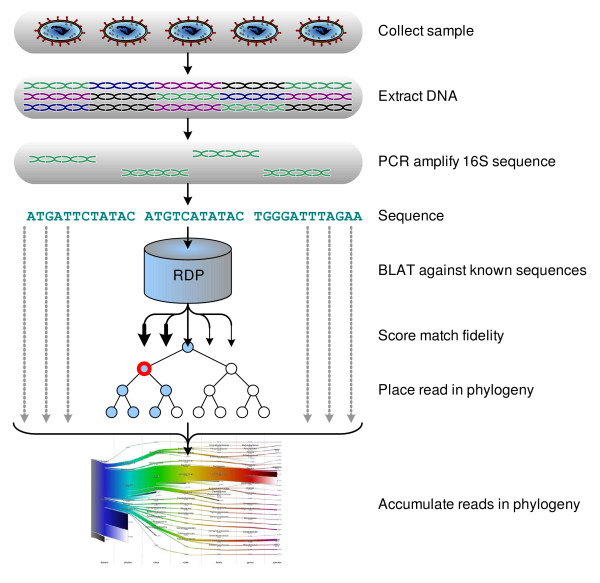
**Methodology overview**. After collecting the bacterial sample, DNA is extracted followed by amplification of 16S rDNA using universal primers. These fragments are then sequenced with high-throughput Pyrosequencing. Each read is queried against a database of known 16S rDNA sequence (mostly obtained from the *Ribosomal Database Project*) using the program *BLAT *and assigned to the most specific and confident node in the phylogeny. Accumulating all the reads in this fashion yields a weighted phylogenetic tree characterizing the bacterial content of the sample.

In this paper, we independently determine for every read the most specific classification within the bacterial phylogeny, and produce a weighted tree that expresses the phylogenetic makeup of the sample. For each read, we use *BLAT*, the BLAST-like alignment tool [18], to search for homology against a database of bacterial 16S rDNA sequences obtained from the *Ribosomal Database Project *[19] and archaeal 16S rDNA sequence from *prokMSA *[20]. We score each resulting homology between the read and a 16S rDNA sequence from the database, filter out weak homologies, and thus produce a set of possible organisms from which the read was obtained. Finally, we assign the read to the most specific location within the phylogeny that includes all these potential organisms (details of this algorithm are described in Methods). By assigning all reads to the phylogeny with the above procedure, we construct a weighted phylogeny representing the 16S rDNA content of the sample. This process is depicted in Figure 1.

Further analysis of bacterial samples involving the translation of read counts to organism concentrations must be undertaken conservatively due to the following caveats. First, there may be an amplification bias of 16S rDNA sequence due to differences in primer annealing preference. Also, variation in 16S rDNA multiplicity in diverse bacterial genomes, among other complications, may result in the over- or under-representation of certain organisms' 16S sequences [18,21].

Our ability to place reads in the phylogeny has two distinct limitations, namely short read-length and unrepresented organisms in the 16S rDNA sequence database. Short read-lengths often lead to high-fidelity matches to multiple 16S sequences in the database. This situation occurs whenever the region from which the read was sampled is highly similar across species of a given genus, family, or even phylum. In this case we are *resolution-limited *in placing a read below a certain depth in the phylogeny. On the other hand, because of the incomplete nature of the 16S rDNA database, a read may not match in its entire length to any known 16S sequence. However, since we believe *a priori *that all reads are derived from amplified 16S rDNA sequences, the closest partial matches of the read to known organisms still allow us to assign the read to the subtree that contains these organisms, although its placement below that level is labeled *unknown*.

### Sample analysis

**Figure 2:**
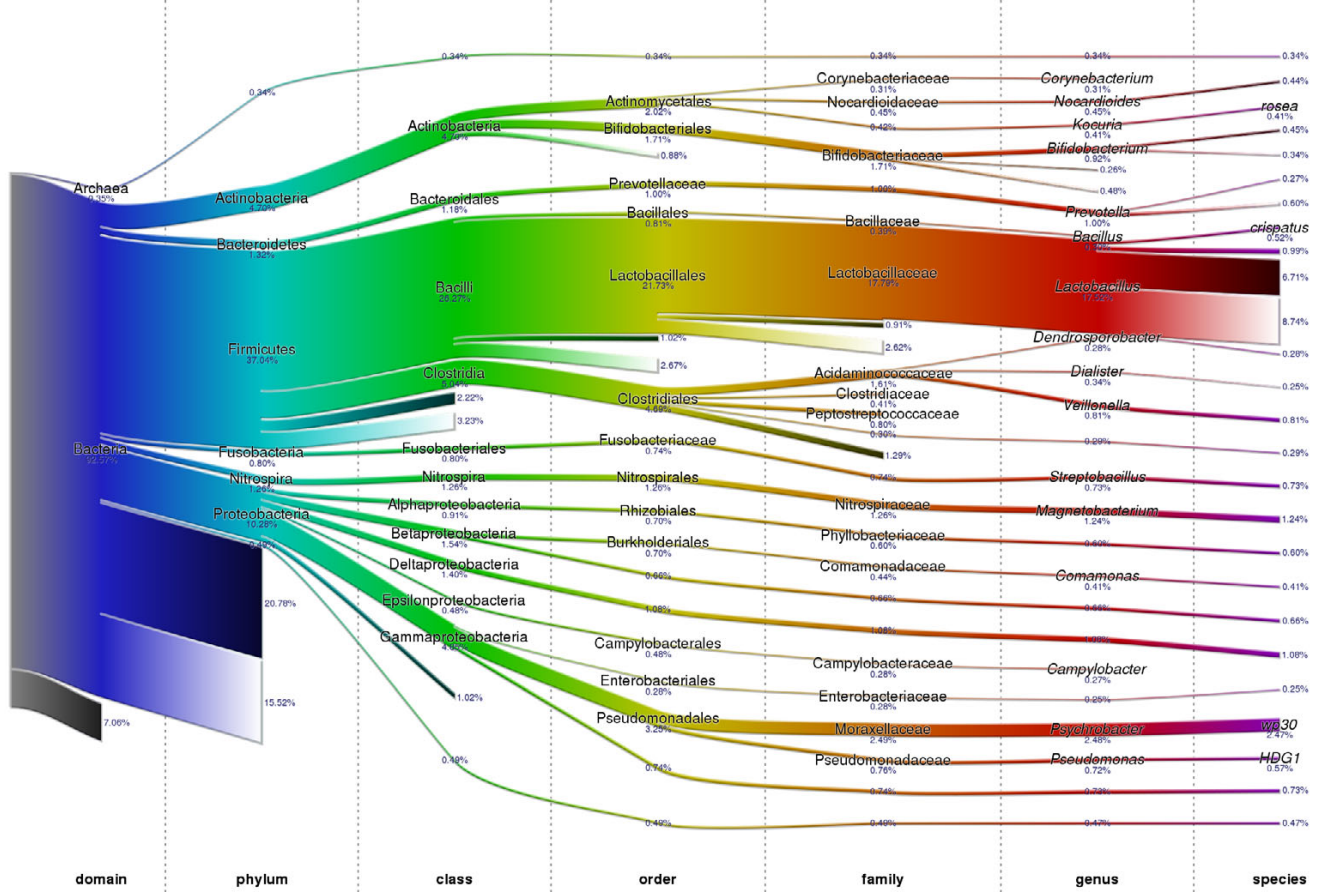
**Combined sample phylogenetic content**. The proposed methodology was applied to the reads obtained from six samples and results were aggregated in a single tree. Branch widths indicate the proportion of reads assigned down those branches in the phylogeny. Branches fading into white represent reads that are resolution limited due to similarity among multiple sub-branches in the phylogeny. Branches fading into black represent reads that do not have full-length homology with any known 16S sequence.

**Figure 3:**
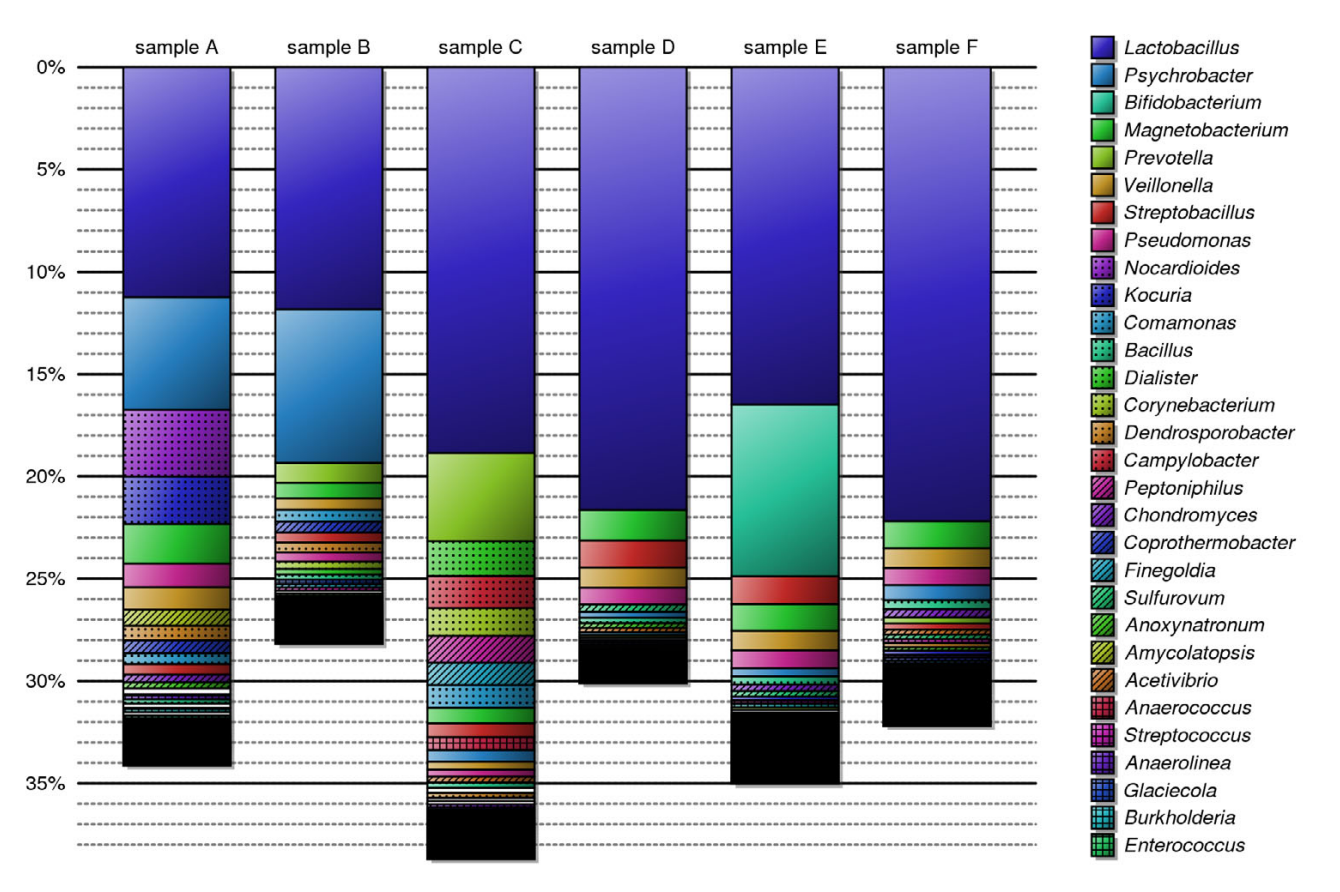
**Top identified genera in samples**. The top 30 genera identified in all six samples are listed in descending order. The percentages of reads in each sample belonging to these genera are indiecated by the height of the bar.

Samples subjected to the above analysis demonstrated substantial overlap with similar studies previously reported [16], as well as significant differences between the samples. Weighted phylogenetic trees obtained from applying our analysis to the six samples are shown in Additional file 2. Figure 2 presents a composite tree generated by accumulating these six trees in equal proportions. Starting from the top, the width of the tree edges represents the proportion of reads that can be confidently placed at that level in the phylogeny. A tree edge that fades into white represents reads that were *resolution-limited *below that level, while reads whose placement is unknown below a particular node are represented by tree edges that fade into black. In Figure 3 we list the top 30 genera discovered in the six samples and identify the proportions of reads belonging to these genera within each sample. Corroborating other studies performed on vaginal bacterial flora, we identified *Lactobacillus *as the dominant genus and detected a significant presence of other genera, including *Psychrobacter*, *Magnetobacterium*, *Prevotella*, *Bifidobacterium*, and *Veillonella *[16]. Aside from the common presence of *Lactobacillus*, each sample exhibited a unique profile of other bacteria, which may be useful in the future for diagnosing abnormal conditions such as vaginosis [5] or predicting the onset of preterm labor [6]. Additional file 3 lists the top 30 genera identified in each sample along with the percentage of reads classified within the genera.

**Figure 4:**
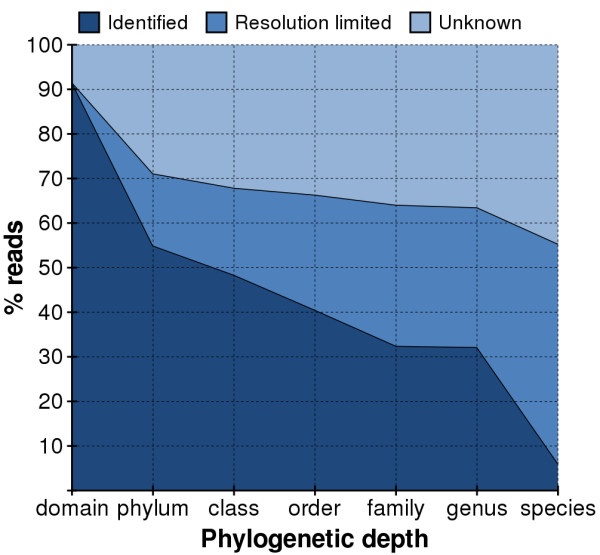
**Read resolution within phylogenetic tree for combined samples**. Combined categorization of the reads in all six samples into their proportion of reads identified (dark blue)/resolution limited (medium)/unknown (light).

**Figure 5:**
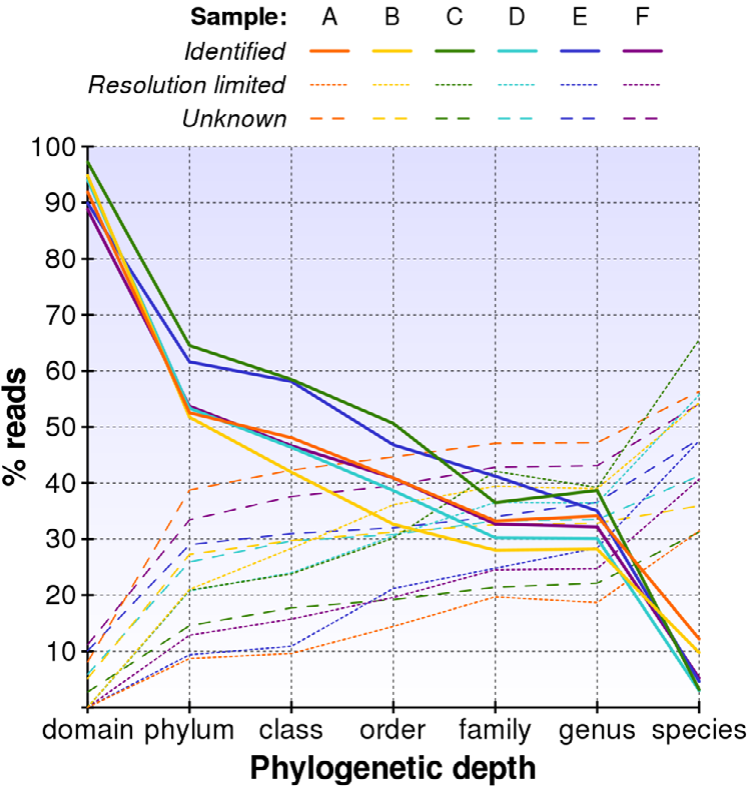
**Read resolution within phylogenetic tree for samples**. Individual categorization of reads in six samples into their proportion of reads identified (solid line)/resolution limited (dotted line)/unknown (dashed line).

In Figure 4 we graph our ability to classify reads into a particular branch at each level of the phylogeny. For those reads that cannot be classified we show the proportion that is *resolution-limited *versus *unknown*. Figure 5 plots these results for each sample separately. Our methodology recognizes 89 – 97% of the reads in each sample as bacterial and fewer than 2% as archaeal; the remaining reads are unrecognizable in our database. While we were able to categorize the genus of 28 – 39% of the reads, only 3 – 12% could be identified with a particular species. Under-representation of 16S rDNA sequence in the database appears to be our dominant limitation in identifying reads at the levels of domain through genus. Fortunately, we expect this limitation to diminish as more 16S rDNA sequences are added to the database. Our ability to identify a particular species, however, is primarily *resolution-limited *due to the overwhelming similarity between species within a genus.

### Effect of read-length

**Figure 6:**
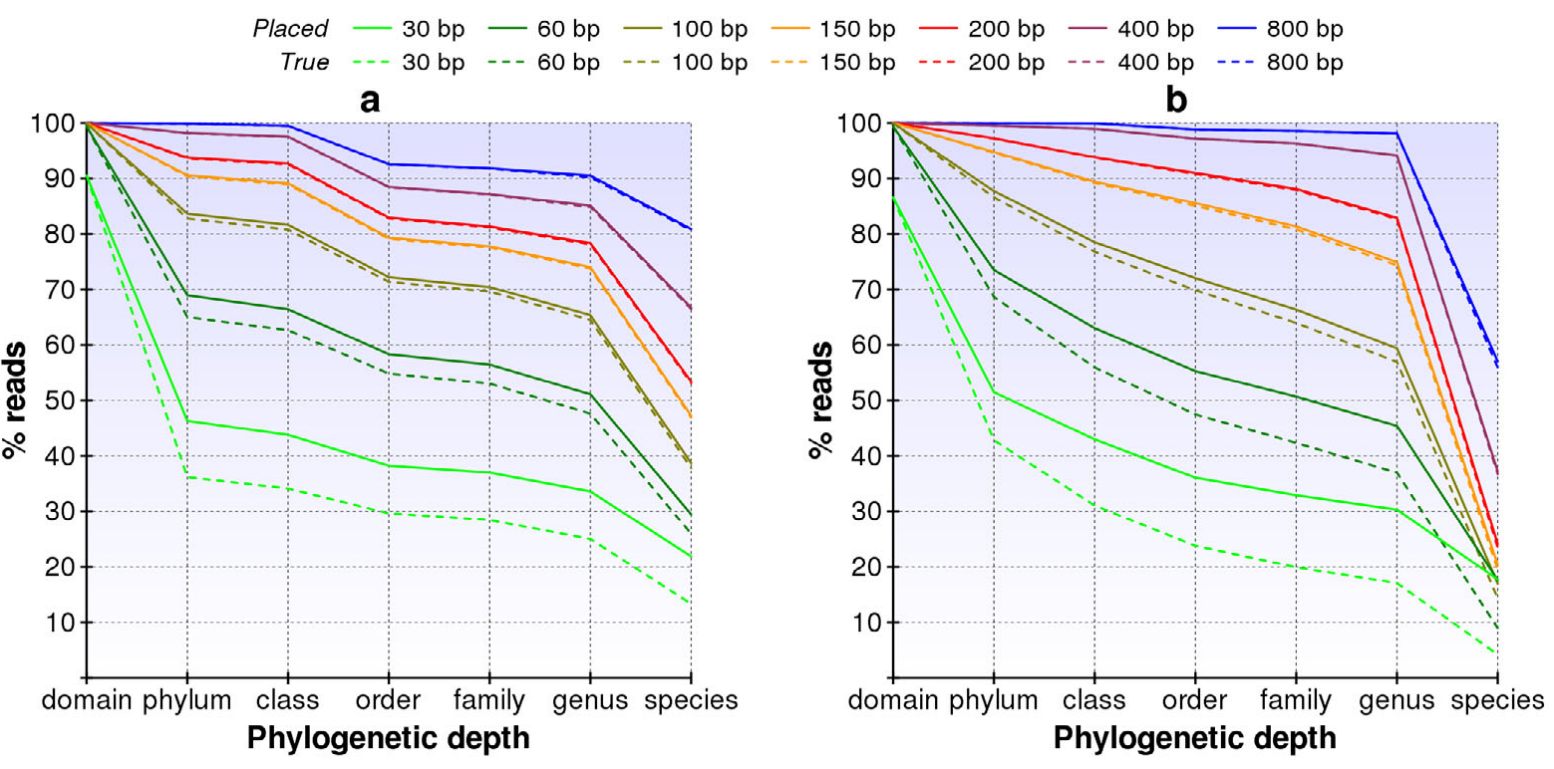
**Simulated read resolution for varying read-lengths in diverse and representative samples**. Simulation results are presented for (a) a diverse set of 387 bacteria and (b) 330 species representative of our samples. Simulated reads had a 20% standard deviation in read-length and a 1% sequencing error rate. Solid lines show assignments made by our methodology, while dashed lines show the proportion that are correctly assigned.

To study the effect of read-length on our ability to place reads in the phylogeny, we simulated the sampling of reads from hypothetical profiles of bacteria for a range of read-lengths from 30 to 800 bp. We analyzed reads sampled from two distinct profiles of bacteria: a *random profile *of 387 diverse bacteria selected from across the entire known phylogenetic tree and a *sample profile *with concentrations of 330 bacteria derived from the analysis of our six samples. The results of applying our methodology to these samples are graphed Figure 6. Solid lines show the proportion of reads that were placed within a particular branch at each level of the phylogeny. Dotted lines with the same color show the proportion of reads that were correctly placed at each level. As both graphs illustrate, read-lengths of 30 and 60 bp are not very effective for discriminating between different bacteria, even at such a broad level as phylum and class.

For the *sample profile *of bacteria our ability to identify genera improves substantially when read-lengths are increased beyond 100 bp due to the high degree of similarity between bacteria in the actual samples we examined (Figure 6b). At 800 bp, we are able to accurately determine almost all of the reads at the genus level, and also correctly determine the species for over half of the reads. As demonstrated in Figure 6b, running the simulation for 100 bp read-lengths closely reproduced the read resolution graph obtained from our six samples, which lends confidence to the stability of the methodology.

For the more diverse, *random profile *of bacteria, the 16S rDNA sequences are sufficiently different that read-lengths greater than 100 bp do not provide much additional benefit (Figure 6a). A read-length of 100 bp, which corresponds to sample data presented here, appears to be competitive with even the longest read-length of 800 bp. Thus, with a very wide diversity of bacteria, it seems that our methodology does not require much greater read-lengths than 100 bp. In practice, however, the sample profile may be more relevant, and therefore longer reads are desirable to improve the resolution of read placement.

There is evidence to suggest that the classification of species within the RDP phylogeny has errors that limit the ability of our methodology to unambiguously classify reads down to the lowest levels of the phylogeny. As an example, suppose we have two species A and B that truly belong to the same genus X, but that species B was misclassified in genus Y. Then, a read that matches both species A and B will be assigned to the family of genera X and Y or an even broader classification. A more accurate database classification will improve the ability of our methodology to identify the genera of the reads.

### Restriction to variable regions

**Figure 7:**
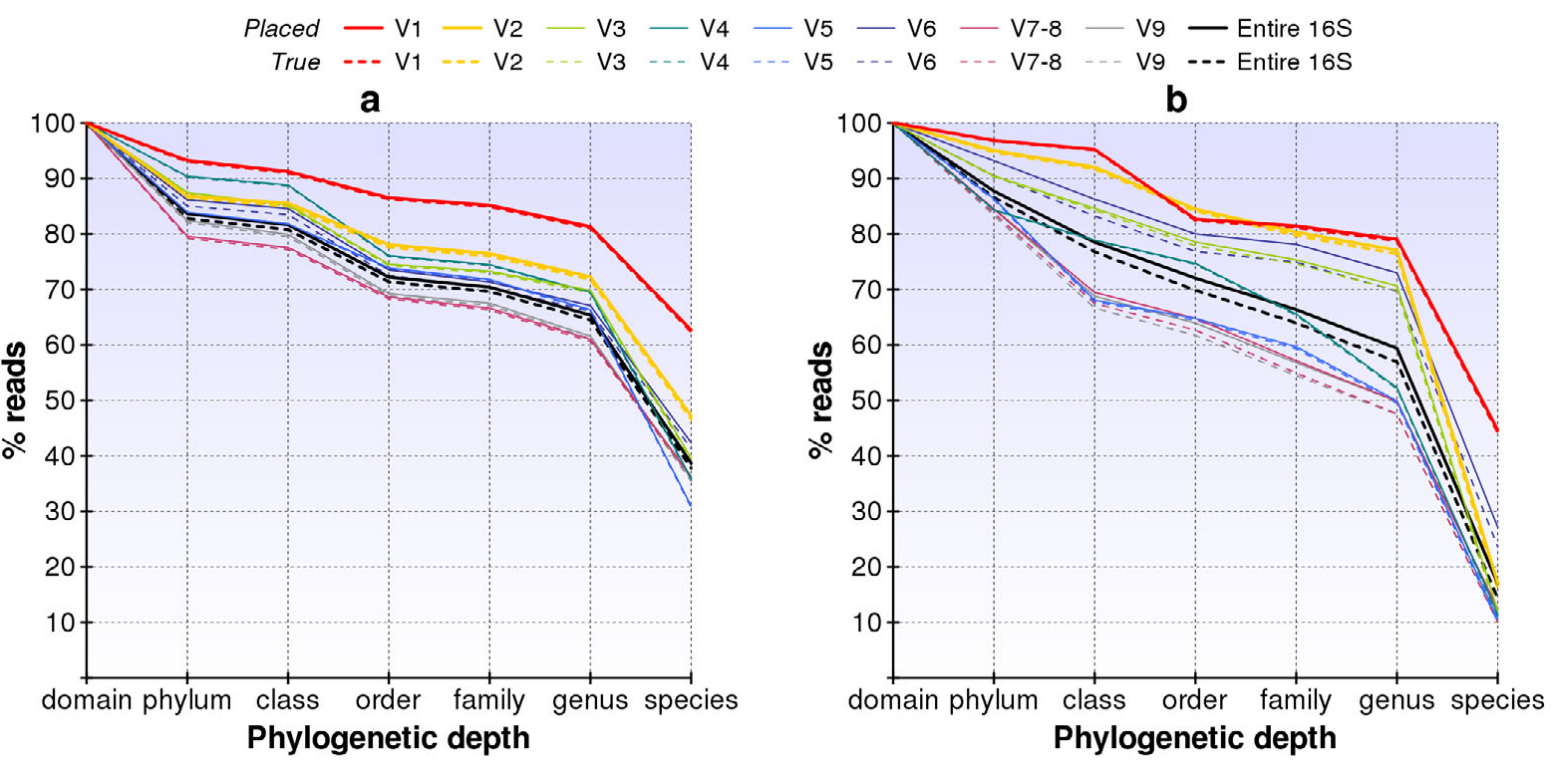
**Simulated read resolution for targeted variable regions in diverse and representative samples**. Simulation results are presented for (a) a diverse set of 387 bacteria and (b) 330 species representative of our samples. Simulated reads had lengths of 100 ± 20 bp and a 1% sequencing error rate. Solid lines show assignments made by our methodology, while dashed lines show the proportion that are correctly assigned.

Our reads often sampled regions in the 16S rDNA that are indistinguishable between species, genera, and even phyla. Restricting the sequencing to short, specific variable regions within the 16S sequence can provide more informative reads [22]. We performed further simulations to assess the effectiveness of such an approach for 100 bp-long Pyrosequencing reads, designing primers for amplifying seven regions each containing one of the 16S rDNA variable regions V1 – V6 and V9, and one region containing V7 and V8 [23]. We describe the construction and verification of the primers in Methods and list the eight amplified regions in Table 1. Figure 7 graphs the read resolution and accuracy results. In both graphs, the bold, black line shows the simulation results when we sample the reads from across the entire 16S rDNA sequence instead of restricting it to a particular variable region.

**Table 1:** 16S variable region range definitions.

**Variable region**	***E. coli *16S rDNA range**			**5' primer**	**3' primer**
			
	**start**	**end**	**length**		
V1	8	120	113	5'-AGAGTTTGATCMTGGCTCAG	5'-TTACTCACCCGTICGCCRCT
V2	101	361	261	5'-AGYGGCGIACGGGTGAGTAA	5'-CYIACTGCTGCCTCCCGTAG
V3	338	534	197	5'-ACTCCTACGGGAGGCAGCAG	5'-ATTACCGCGGCTGCTGG
V4	519	806	288	5'-TGCCAGCAGCCGCGGTAA	5'-GGACTACARGGTATCTAAT
V5	787	926	140	5'-ATTAGATACCYTGTAGTCC	5'-CCGTCAATTCMTTTGAGTTT
V6	907	1073	167	5'-AAACTCAAAKGAATTGACGG	5'-ACGAGCTGACGACARCCATG
V7 & VV8	1054	1406	353	5'-CATGGYTGTCGTCAGCTCGT	5'-ACGGGCGGTGTGTAC
V9	1392	1507	116	5'-GTACACACCGCCCGT	5'-TACCTTGTTACGACTT

Regions were chosen to be mostly non-overlapping, each containing one or two variable regions. Coordinates are given relative to the 1542 bp *E. coli *K12 16S rDNA sequence.

For the *random profile *of bacteria we could slightly improve our resolving power by restricting reads to shorter variable regions, particularly with region V1 (Figure 7a). For the more realistic *sample profile*, by choosing the appropriate variable region we could improve results dramatically and achieve a resolution similar to 150 – 200 bp reads sampled from across the entire 16S gene. When reading from region V1 we were able to identify the genus of 79% of the reads instead of 57% when sampling across the entire 16S sequence and 44% of the species instead of only 14% (Figure 7). Region V2 was best able to determine the classification for the reads at the level of order, correctly identifying 84% of the reads compared to 70% when sampling from across the entire 16S gene. Thus, our study suggests that identifying the phylogenetic content of bacterial communities with short reads will be best achieved by targeting variable regions that are specifically chosen for each class of bacterial environment.

## Conclusion

By combining high-throughput Pyrosequencing with a novel analysis methodology, we identified phylogenies of bacteria present in the human vagina during pregnancy. Previous studies of the correlation between identified bacteria and preterm labor, and attempts to treat such microorganisms have produced conflicting results [24-26]. Our methodology for studying in-depth the ecology of human pregnancy will assist in understanding the correlation between vaginal microorganisms and complications in pregnancy.

Our simulations indicate that the methodology is currently limited by two factors: short read-lengths of Pyrosequencing and the incomplete nature of 16S rDNA databases. As more bacteria are sequenced and added to the database, the effects of the second limitation will decrease. Improvements in sequencing technology will increase read-lengths and enhance our ability to distinguish between genera. In order to best identify particular species, using our methodology we can identify and isolate the most informative 16S variable region.

## Methods

### Identifying 16S sequence

The first stage of our analysis matches reads against known 16S rDNA sequences, or finds the closest matches to known organisms. We leverage the *Ribosomal Database Project *release 9 update 39 for its catalog of bacteria and their phylogenetic relationships [19] and the *prokMSA *database as a representative set of archaeal sequence [20]. For each read, we used the tool *BLAT *(BLAST-Like Alignment Tool) [18] to quickly identify matches between reads and the combined bacterial/archaeal database, using a minimum identity of 90% and a minimum match/mismatch score of 15 bases.

To score the homology between a read and a database sequence, we approximate the probability that the read came from an organism with a *p *= 98% sequence similarity to the given read as follows:

$$P\left(\text{related}\right)=\prod_{i=1}^{L}\left[M_{i}pQ_{i}+\left(1-M_{i}\right)\left(1-pQ_{i}\right)\right],$$

where *M*_*i *_are indicator variables that are 1 if position *i *in the read matches with the database sequence in their alignment and 0 otherwise. The variables *Q*_*i *_are the probabilities that the read bases were called correctly, derived from the sequence quality scores.

Then, to judge whether or not we believe a read came from the organism's phylotype, we compare this probability against the probability for a hypothetical read that falls at the boundary of similarity

*P *(related limit) = *p*^*pL *^(1 - *p*)^(1 - *p*) *L*^.

Reads that score above this probability limit are classified as *known*, while reads that score below the limit are classified as *unknown*.

### Placing reads in the phylogeny

Each read that results in a *known *match will typically also match with many additional organisms. For example, a read may match with several species within the same genus, in which case we cannot identify the exact species of the read. However, if all the *known *hits at least fall within the same genus then we are confident the read was sampled from an organism belonging to that genus. In this way, for each read our goal is to determine the most specific classification within the phylogeny that likely contains the organism from which the read was obtained.

**Figure 8:**
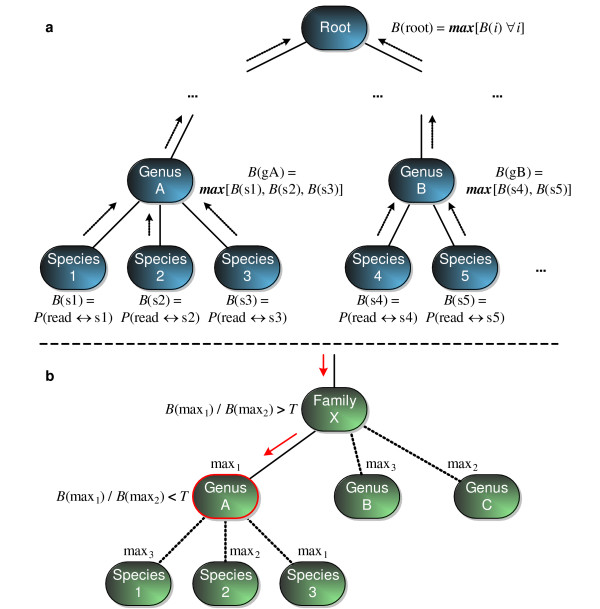
**Placing read in phylogeny**. Computing the most specific and confident placement of a read in the phylogenetic tree occurs in two stages. First, for each internal node we compute a score that is equal to the maximum of the scores of its children. Second, we traverse down the tree from the root until we find a node for which the child with the second maximum score is within a threshold *T *of the maximum score, or until we reach a leaf node.

We analyze each read *r *using the following alrogithm. For every node *i *in the phylogenetic tree, we assign a value *B*(*i*) as follows. For leaf nodes, we set *B*(*i*) = *P*(*r *related to *i*) defined above for organisms with a scored BLAT hit, and *B*(*i*) = 0 otherwise. For internal nodes, we set *B*(*i*) = max_*j *∈ children (*i*) _*B*(*j*). This process is illustrated in Figure 8a. At the root node we will therefore have *B*(root) = max_*i *_*P*(*r *related to *i*).

Next, we traverse down the tree starting at the root node. At each internal node, if the ratio of the two maximal child nodes *j *and *k *exceeds a threshold *T *(i.e. *B*(*j*)/*B*(*k*) > *T*), or if *B*(*j*) is the only non-zero child, then we descend to node *j *and repeat the procedure. Once the procedure terminates in an internal node or a leaf node *i*, we believe with a confidence level related to *T *that the read came from an organism belonging to the subtree rooted at *i*. An example of this is illustrated in Figure 8b. We experimented with the choice of *T *over several orders of magnitude and found that the resulting analysis varied only very slightly. For the analyses performed in this study, we used *T *= 0.01.

### Simulating reads

For our analysis of read-length and variable region resolving power, we simulated reads from two hypothetical collections of species. The *random profile *consists of 387 species of bacteria, selected by randomly traversing down the phylogenetic tree from the root to a leaf, picking each branch with uniform probability, resulting in very high diversity. The *sample profile *constitutes 330 species of bacteria, created by sampling species from a distribution of genera and species that was consistent with the analysis results from our six samples.

For each simulation, a read simulator generated reads sampled from 16S rDNA sequence selected randomly according to either the *random *or the *sample profile*. Reads were sampled with uniform probability from across the rDNA sequence, with a read-length drawn from a Gaussian distribution with average read-lengths of 30, 60, 100, 150, 200, 400, or 800 bp and standard deviations of 20%. Sequencing errors were introduced into the reads at a rate of 1% that consisted of mutations, insertions, deletions, and homopolymer run count errors characteristic of Pyrosequencing.

To understand the effect of read-length on the resolving power of our methodology, we simulated reads from both the *random *and *sample profile *with the seven read-lengths *L *of 30 – 800 bp. For each of the 2 × 7 = 14 parameter sets we produced 30 Mb of simulated read data (*N *reads @ *L *bp = 30 · 10^6 ^bp) and applied our analysis. By annotating the source species for each read we able to measure the accuracy of its placement in the phylogenetic tree, as in Figure 6.

To study the effectiveness of restricting the sequencing to known variable regions, we first selected a set of eight minimally-overlapping regions within the 16S rDNA sequence: seven regions each contained one of the known variable regions V1 – V6 and V9, and one region contained both V7 and V8 [27]. These regions are listed in Table 1 with their *E. coli *16S rDNA sequence coordinate ranges as well as 5' and 3' broad-range amplification primers, which we validated by PCR amplifying 16 rDNA from *E. coli*. For each primer pair we performed 15 cycles of touch-down PCR, going from 94°C for 30 s, to an annealing temperature ranging from 70°C to 50°C for 30 s, and finally extending at 72°C for 30 s. We then performed 30 additional cycles at 94°C for 45 s, 50°C for 45 s, and 72°C for 45 s, and verified the resulting products via gel electrophoresis.

Next, we again produced sets of simulated reads for both the *random *and the *sample profile*, restricted to each region, for a total of 2 × 8 = 16 sets. Each read data set consisted of 300,000 reads with an average read-length of 100 bp and a 1% error rate. We applied our analysis and measured the accuracy of read placement in the phylogeny in Figure 7.

## Authors' contributions

AS, S Batzoglou, and MR designed the analysis methodology. AS implemented the methodology and conducted the analysis. S Bigdeli, RJ, and MR performed the sample preparation and sequencing. MLD, SW, KMP, YYEL, and MMT collected the samples. AS, YYES, S Batzoglou, and MR wrote the manuscript. All authors read and approved the final manuscript.

## Supplementary Material

Additional File 1 **Sequencing statistics for six samples**. This spreadsheet provides statistics for the sequence read data obtained for the six samples. Click here for file

Additional File 2 **Weighted phylogenetic trees for six samples**. The images represent weighted phylogenetic trees resulting from the application of our analysis to each of the six samples. Click here for file

Additional File 3 **Top genera identified in six samples**. This spreadsheet lists the top 30 genera identified in each sample along with their proportion of reads. Click here for file

## References

[B1] Amann RI, Ludwig W, Schleifer KH (1995). Phylogenetic identification and in situ detection of individual microbial cells without cultivation. Microbiol Rev.

[B2] Rappé MS, Giovannoni SJ (2003). The Uncultured Microbial Majority. Annu Rev Microbiol.

[B3] Tringe SG, Rubin EM (2005). Metagenomics: DNA sequencing of environmental samples. Nat Rev Genet.

[B4] Anderson BE, Dawson JE, Jones DC, Wilson KH (1991). *Ehrlichia chaffeensis*, a new species associated with human ehrlichiosis. J Clin Microbiol.

[B5] Verhelst R, Verstraelen H, Claeys G, Verschraegen G, Delanghe J, Van Simaey L, De Ganck C, Temmerman M, Vaneechoutte M (2004). Cloning of 16S rRNA genes amplified from normal and disturbed vaginal microflora suggests a strong association between *Atopobium vaginae, Gardnerella vaginalis *and bacterial vaginosis. BMC Microbiol.

[B6] Goldenberg RL, Hauth JC, Andrews WW (2000). Intrauterine Infection and Preterm Delivery. N Engl J Med.

[B7] Gravett MG, Novy MJ, Rosenfeld RG, Reddy AP, Jacob T, Turner M (2004). Diagnosis of intra-amniotic infection by proteomic profiling and identification of novel biomarkers. JAMA.

[B8] Sbarra AJ, Selvaraj RJ, Cetrulo CL, Feingold M, Newton E, Thomas GB (1985). Infection and phagocytosis as possible mechanisms of rupture in premature rupture of the membranes. Am J Obstet Gynecol.

[B9] McGregor JA, Lawellin D, Franco-Buff A, Todd JK, Makowski EL (1986). Protease production by microorganisms associated with reproductive tract infection. Am J Obstet Gynecol.

[B10] Andrews WW, Hauth JC, Goldenberg RL, Gomez R, Romero R, Cassell GH (1995). Amniotic fluid interleukin-6: correlation with upper genital tract microbial colonization and gestational age in women delivered after spontaneous labor versus indicated delivery. Am J Obstet Gynecol.

[B11] Fortunato SJ, Menon RP, Swan KF, Menon R (1996). Inflammatory cytokine (interleukins 1, 6 and 8 and tumor necrosis factor-alpha) release from cultured human fetal membranes in response to endotoxic lipopolysaccharide mirrors amniotic fluid concentrations. Am J Obstet Gynecol.

[B12] Fortunato SJ, Menon R, Lombarda SJ (2002). Role of tumor necrosis factor-alpha in the premature rupture of membranes and preterm labor pathways. Am J Obstet Gynecol.

[B13] Margulies M, Egholm M, Altman WE, Attiya S, Bader JS, Bemben LA, Berka J, Braverman MS, Chen YJ, Chen Z, Dewell SB, Du L, Fierro JM, Gomes XV, Godwin BZC, He W, Helgesen S, Ho CH, Irzyk GP, Jando SC, Alenquer MLI, Jarvie TP, Jirage KB, Kim J-B, Knight JR, Lanza JR, Leamon JH, Lefkowitz SM, Lei M, Li J, Lohman KL, Lu H, Makhijani VB, McDade KE, McKenna MP, Myers EW, Nickerson E, Nobile JR, Plant R, Puc BP, Ronan MT, Roth GT, Sarkis GJ, Simons JF, Simpson JW, Srinivasan M, Tartaro KR, Tomasz A, Vogt KA, Volkmer GA, Wang SH, Wang Y, Weiner MP, Yu P, Begley RF, Rothberg JM (2005). Genome sequencing in microfabricated high-density picolitre reactors. Nature.

[B14] Rogers YH, Venter JC (2005). Genomics: massively parallel sequencing. Nature.

[B15] Ronaghi M, Karamohamed S, Petterson B, Uhlen M, Nyren P (1996). Real-time DNA sequencing using detection of pyrophosphate release. Anal Biochem.

[B16] Hyman RW, Fukushima M, Diamona L, Kumm J, Giudice LC, Davis RW (2005). Microbes on the human vaginal epithelium. Proc Natl Acad Sci.

[B17] Sogin ML, Morrison HG, Huber JA, Welch DM, Huse SM, Neal PR, Arrieta JM, Herndl GJ (2006). Microbial diversity in the deep sea and the underexplored "rare biosphere". Proc Natl Acad Sci.

[B18] Kent WJ (2002). BLAT – the BLAST-Like Alignment Tool. Genome Res.

[B19] Cole JR, Chai B, Farris RJ, Wang Q, Kulam SA, McGarrell DM, Garrity GM, Tiedje JM (2005). The Ribosomal Database Project (RDP-II): sequences and tools for high-throughput rRNA analysis. Nucleic Acids Res.

[B20] DeSantis TZ, Dubosarskiy I, Murray SR, Andersen GL (2003). Comprehensive aligned sequence construction for automated design of effective probes (CASCADE-P) using 16S rDNA. Bioinformatics.

[B21] Von Wintzingerode F, Gobel UB, Stackebrandt E (1997). Determination of microbial diversity in environmental samples: pitfalls of PCR-based rRNA analysis. FEMS Microbiol Rev.

[B22] Monstein H, Nikpour-Badi S, Jonasson J (2001). Rapid molecular identification and subtyping of *Helicobacter pylori *by Pyrosequencing of the 16S rDNA variable V1 and V3 regions. FEMS Microbiol Lett.

[B23] Gray MW, Sankoff D, Cedergren RJ (1984). On the evolutionary descent of organisms and organelles: a global phylogeny based on a highly conserved structural core in small subunit ribosomal RNA. Nucleic Acids Res.

[B24] Carey JC, Klebanoff MA, Hauth JC, Hillier SL, Thom EA, Ernest JM, Heine RP, Nugent RP, Fischer ML, Leveno KJ, Wapner R, Varner M, Trout W, Moawad A, Sibai BM, Miodovnik M, Dombrowski M, O'Sullivan MJ, Van Dorsten JP, Langer O, Roberts J (2000). Metronidazole to prevent preterm delivery in pregnant women with asymptomatic bacterial vaginosis. N Engl J Med.

[B25] Klebanoff MA, Carey JC, Hauth JC, Hillier SL, Nugent RP, Thom EA, Ernest JM, Heine RP, Wapner RJ, Trout W, Moawad A, Miodovnik M, Sibai BM, Van Dorsten JP, Dombrowski MP, O'Sullivan MJ, Varner M, Langer O, McNellis D, Roberts JM, Leveno KJ (2001). Failure of Metronidazole to Prevent Preterm Delivery among Pregnant Women with Asymptomatic *Trichomonas vaginalis *Infection. N Engl J Med.

[B26] Thinkhamrop J, Hofmeyr GJ, Adetoro O, Lumbiganon P (2002). Prophylactic antibiotic administration in pregnancy to prevent infectious morbidity and mortality. Cochrane Database Syst Rev.

[B27] Neefs J, Van de Peer Y, De Rijk P, Goris A, De Wachter R (1991). Compilation of small ribosomal subunit RNA sequences. Nucleic Acids Res.

