# Supplementary material for: Bacterial flora-typing with targeted, chip-based Pyrosequencing
Source: BMC Microbiol. 2007 Nov 30;7:108. doi: 10.1186/1471-2180-7-108 (PMC2244631; doi:10.1186/1471-2180-7-108)
Supplement: Additional File 2 — Weighted phylogenetic trees for six samples. The images represent weighted phylogenetic trees resulting from the application of our analysis to each of the six samples. [file 1471-2180-7-108-S2.pdf]

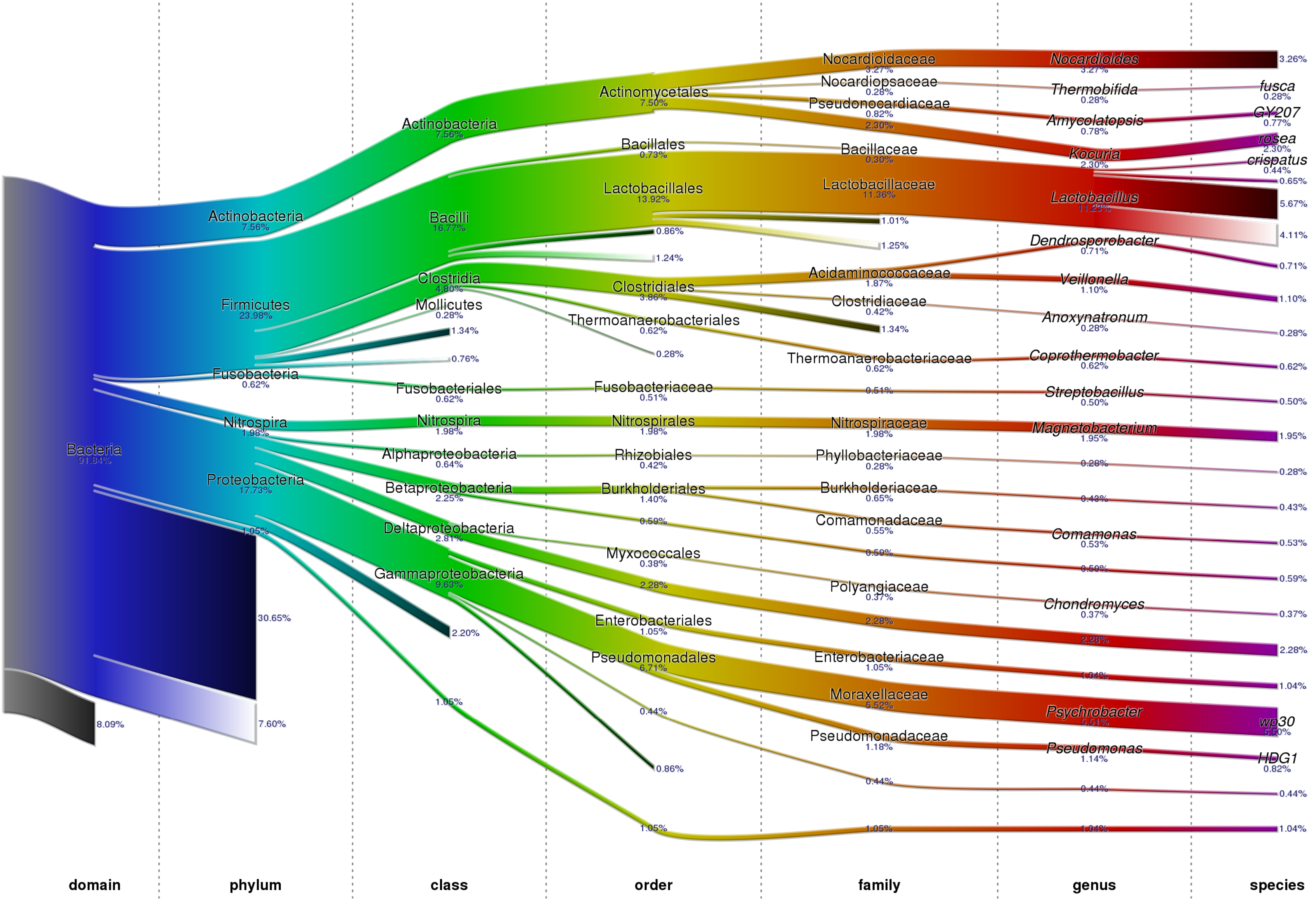

Supplementary Figure S1. *Sample A phylogenetic content.*

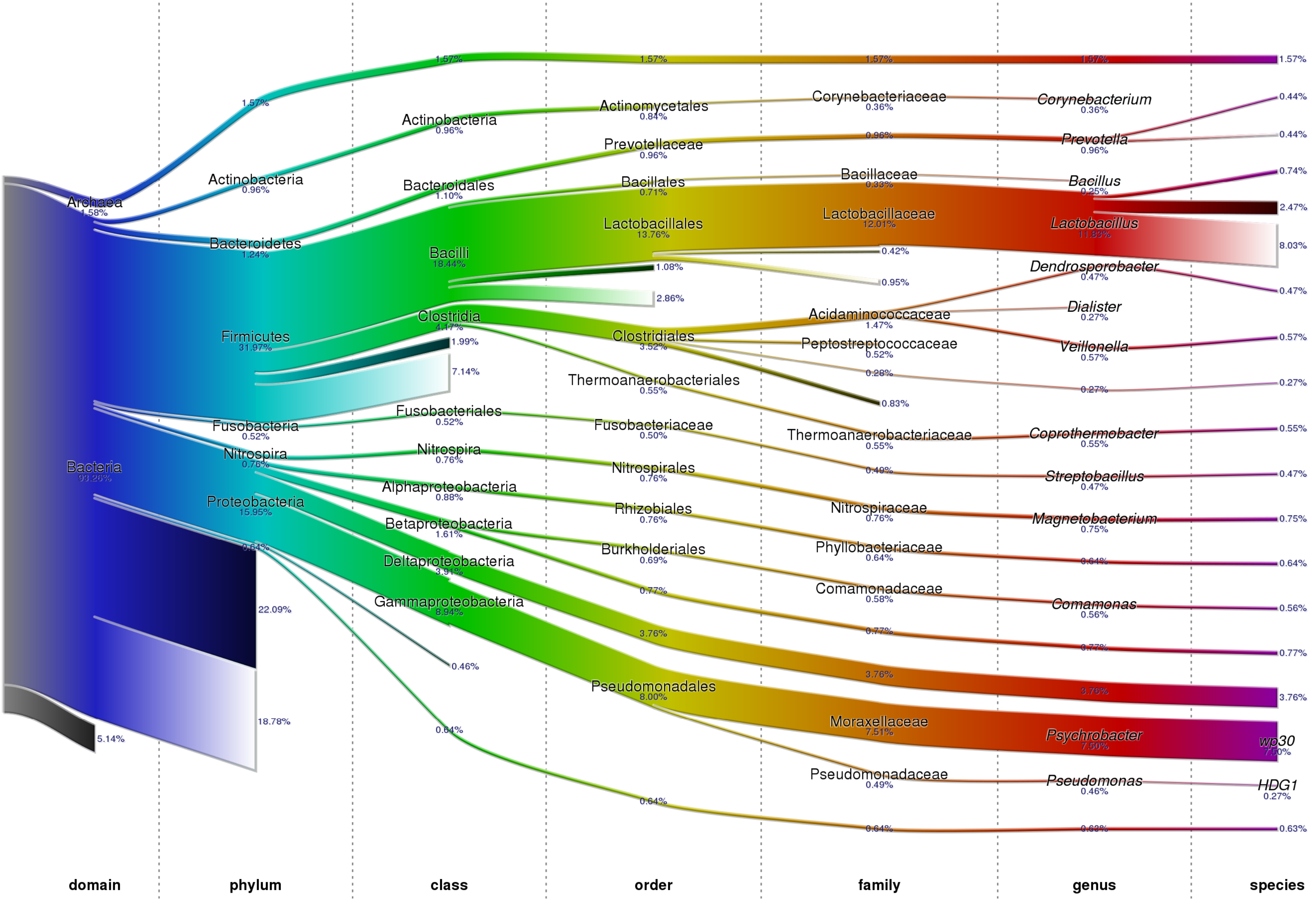

Supplementary Figure S2. *Sample B phylogenetic content.*

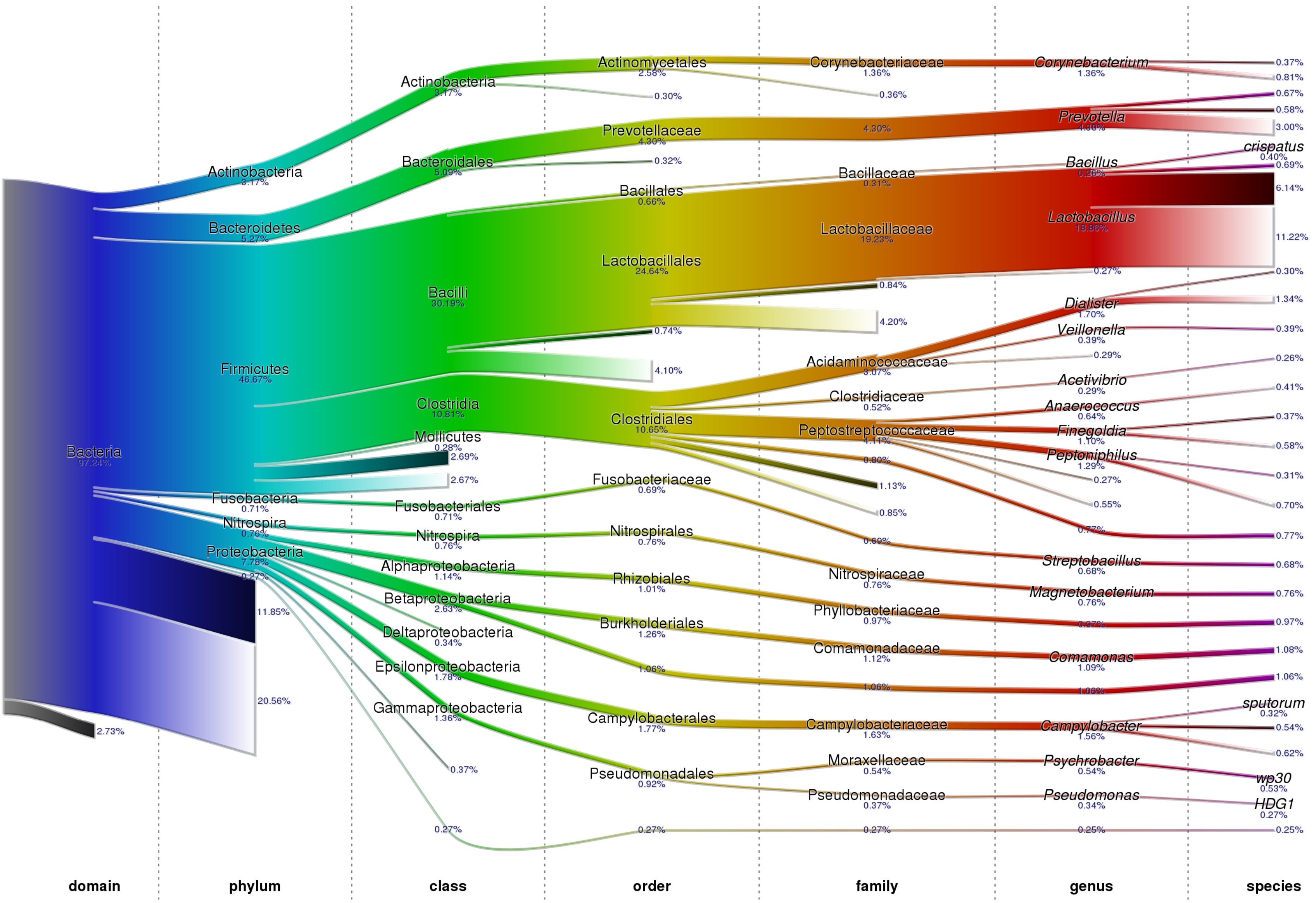

Supplementary Figure S3. *Sample C phylogenetic content.*

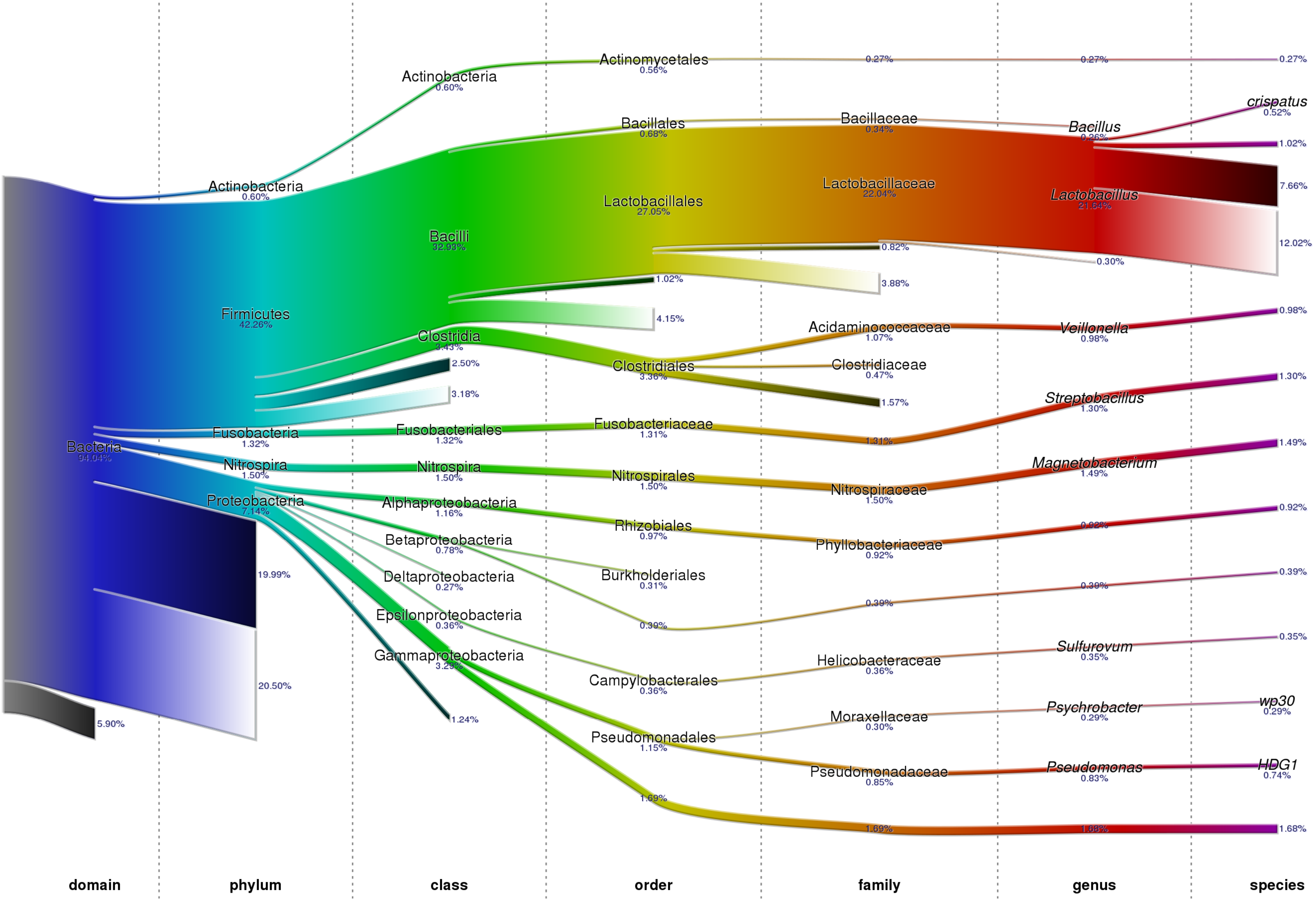

Supplementary Figure S4. *Sample D phylogenetic content.*

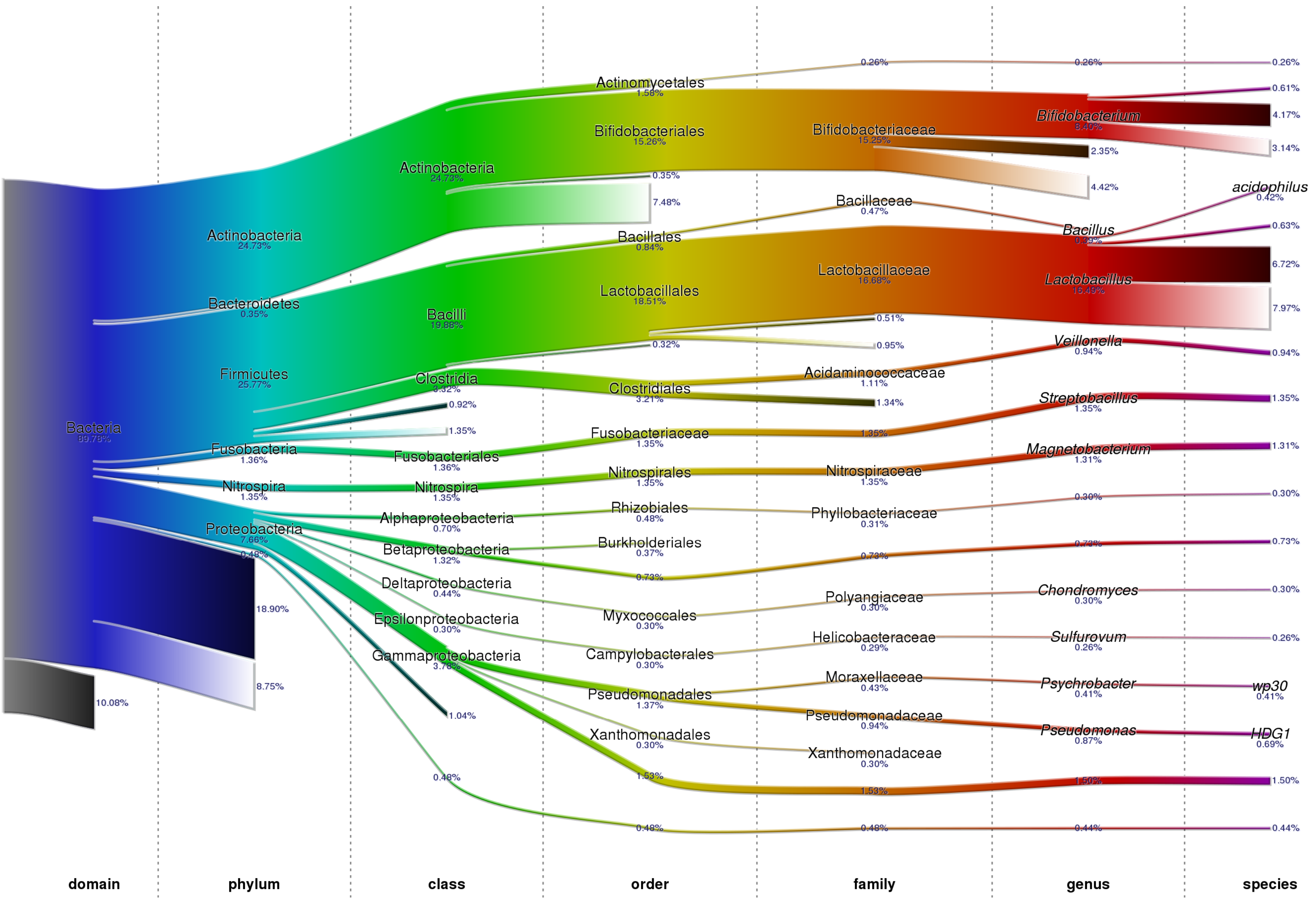

Supplementary Figure S5. *Sample E phylogenetic content.*

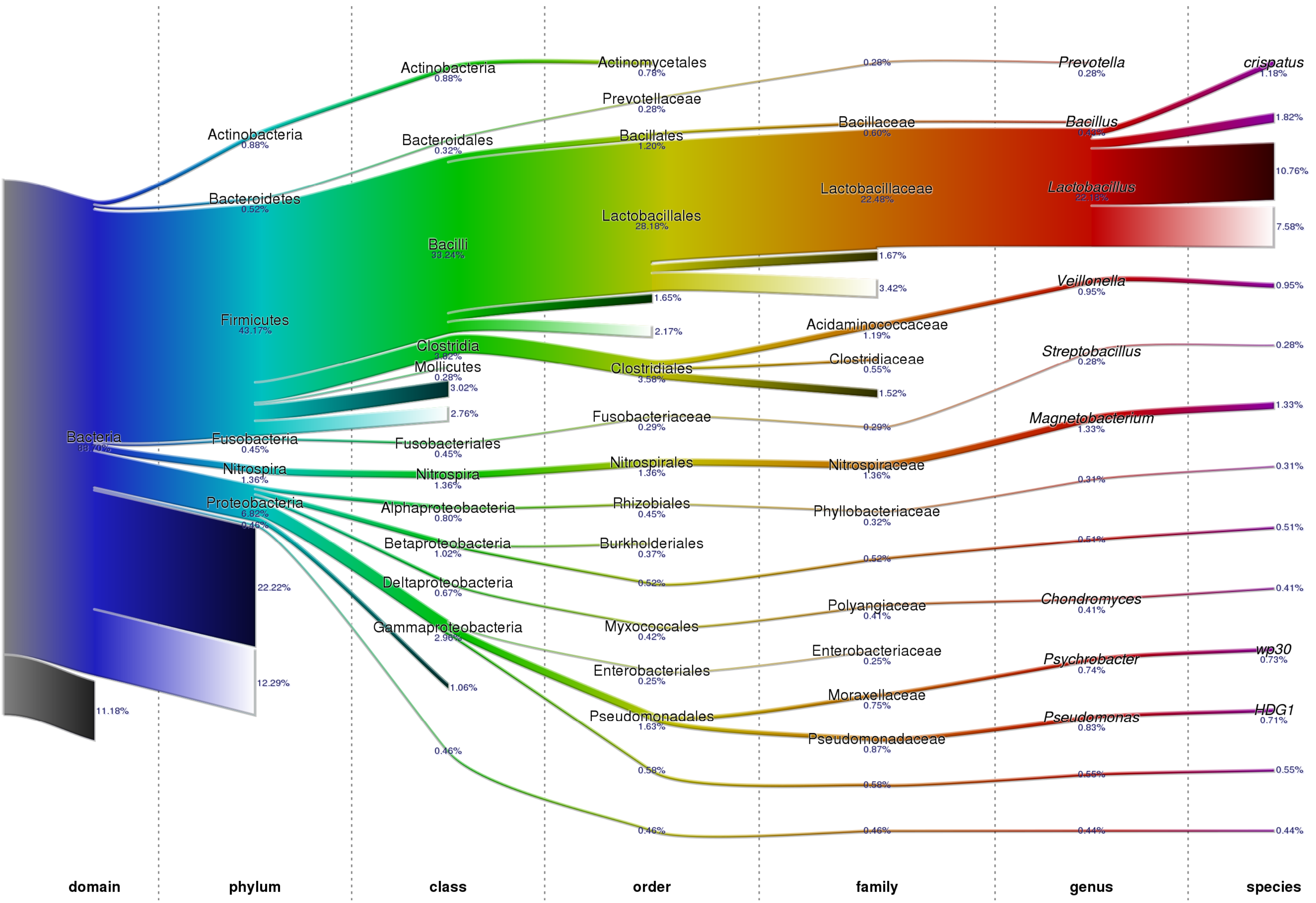

Supplementary Figure S6. *Sample F phylogenetic content.*
